# Incubation and water temperatures influence the performances of loggerhead sea turtle hatchlings during the dispersal phase

**DOI:** 10.1038/s41598-018-30347-3

**Published:** 2018-08-09

**Authors:** Shohei Kobayashi, Nanamo Aokura, Ryohei Fujimoto, Keisuke Mori, Yoshinori Kumazawa, Yusuke Ando, Tsuyoshi Matsuda, Hiroshi Nitto, Katsuhiko Arai, Gen Watanabe, Tomomi Saito

**Affiliations:** 1 Department of Biological Production Science, United Graduate School of Agricultural Science, Tokyo University of Agriculture and Technology, Tokyo, 183-8509 Japan; 2 Toba Aquarium, Mie, 517-8517 Japan; 3 Usa Marine Biological Institute, Kochi University, Kochi, 781-1164 Japan; 4 Haruno-cho, Kochi, 781-0315 Japan; 5 Port of Nagoya Public Aquarium, Aichi, 455-0033 Japan; 6 Laboratory of Veterinary Physiology, Institute of Agriculture, Tokyo University of Agriculture and Technology, Tokyo, 183-0054 Japan

## Abstract

Artificial manipulation of incubation temperature has been proposed as a potential strategy for mitigating the effects of climate change on sea turtles for which sex determination is temperature-dependent, but thermal manipulation may also affect hatchling survival. Here, we demonstrated that incubation and water temperatures influenced several performance traits that contribute to the survival of loggerhead sea turtles (*Caretta caretta*) during the post-hatchling dispersal phase. Hatchlings from warm incubation temperatures (31 °C) had significantly shorter incubation periods, higher initial swimming performance, lower sustained swimming performance, and lower growth rates during the first three weeks post-hatching, as well as higher blood glucose concentrations, than those from cool incubation temperatures (27.5 °C). Hatchlings in warm water temperatures (30 °C) exhibited significantly greater swimming performance than those in cool water temperatures (27 °C). Our results indicated that altering incubation temperatures indirectly influences the survival of loggerhead hatchlings by modifying their swimming performance and growth rates, which may affect hatchling predator-avoidance capability. Moreover, thermal manipulation may alter the incubation period, exposing hatchling to water temperatures that they would not otherwise normally experience, which may affect swimming performance. Our results suggest that such conservation strategies may influence their survival, and thus should be carefully considered.

## Introduction

Climate change is recognized as one of the worldwide environmental problems. According to the Fifth Assessment Report of the Intergovernmental Panel on Climate Change, global ambient temperatures will increase by 1.0–3.7 °C during the 21st centuriy^1^. Thus, the effect of this environmental problem on the sex ratio of animals with temperature-dependent sex determination (TSD), whose sex is determined by the incubation temperature (IT), is of growing concern^2–9^. Sea turtle species possess TSD, with warmer ITs producing females^10^, and as such, the increasing feminisation of sea turtle population due to climate change has been suggested^2–6^. To prevent the production of single sex populations, artificial manipulations of ITs, for example, through the provision of shade and watering, are currently considered to be potentially viable conservation strategies for sea turtles^5,7–9^.

Following emergence from the nests, sea turtle hatchlings swim vigorously (frenzy swimming) offshore for approximately one day^11^, and then leave the neritic zone for several weeks (post-frenzy period)^12,13^. Given the high predation pressure during these frenzy and post-frenzy periods, larger body sizes and superior locomotor performances may enhance hatchling predator avoidance, and thus, larger and better performing hatchlings have a greater likelihood of surviving these critical periods^14^. Notably, among TSD reptiles, IT was reported to affect not only sex determination but also body size and locomotor performance^15^, which are likely to influence survival. As such, artificial manipulation of IT may alter not only their sex ratio but could also influence hatchling survival at early life stages. In addition, Booth and Evans^16^ reported that the swimming performance of green sea turtle hatchlings (*Chelonia mydas*) during the frenzy period was higher in warm water temperature (WT) than that in cool WT. Because IT influences the incubation period^17,18^, artificial manipulation of IT may therefore expose hatchlings to water temperatures that they would not otherwise normally experience. From this point of view, thermal manipulation might indirectly influence hatchling swimming performances. Thus, a better understanding of the effects of IT and WT on hatchling performance traits, and consequently their survival, is important for the conservation of sea turtle species.

Several studies have demonstrated the effect of nest IT on performance traits in sea turtles that may affect hatchling survival during the post-hatchling dispersal period. With regard to size, incubation periods are prolonged as a result of cooler IT, and consequently the period when the yolk is converted to tissue is also prolonged; as a result, hatchlings incubated under cooler IT condition tend to be larger^19^. In terms of swimming performance, previous studies have focused exclusively on the frenzy period (<24 h)^16,19–24^. However, because it takes at least several days for hatchlings to leave the neritic zone^12^, the effect of IT on sustained swimming performance during the post-frenzy period should also be assessed. Furthermore, during the post-frenzy period, hatchlings presumably swim fuelled by energy derived from the remaining yolk. Because embryos consume a great amount of energy during incubation, extended incubation period as a result of cooler IT reduces the amount of yolk available to hathclings^19,25^. Consequently, hatchlings may have fewer energy reserves to draw from during the post-frenzy period when incubated under cooler IT conditions, but, quantitative experimental to explore this issue are lacking.

Hatchlings growth and development depends on nutrients in the remaining yolk and subsequent foraging during the post-frenzy period^26^. Hatchlings that grow more rapidly experience a shorter period of time when they are vulnerable to predation by gape-limited predators, and growth rate is therefore likely to affect hatchling survival^27^. For loggerhead sea turtle (*Caretta caretta*) hatchlings in the North Atlantic, growth rates differ depending on latitude and nesting season^27^, suggesting that environmental factors like IT might play a role in determination of the growth rate, but this has yet to be experimentally examined.

The use of incubators is a powerful tool for assessing the effects of IT on hatchling performance because conditions (e.g., temperatures) can be controlled, and genetic and maternal factors can be taken into account by dividing the clutch. In the present study, we divided clutches of loggerhead sea turtles into different incubators with the objective of examining the effect of IT on size, growth rate, and swimming performance, and the effect of WT on swimming performance. In addition, to determine whether IT affects the remaining energy of hatchlings during the post-frenzy period, we measured blood glucose concentration as a representative indicator of energy sources.

## Results

### Hatchlings

We used three clutches of loggerhead sea turtles in the experiments. These clutches were divided into two groups, and incubated at either 31 °C (warm IT) or 27.5 °C (cool IT). These ITs represent the mean male-producing temperature (MPT) and female-producing temperature (FPT) at major rookeries of loggerhead sea turtles in the North Pacific, our field study area^18^. After four days post hatching, we measured straight carapace length (SCL, mm), straight carapace width (SCW, mm), and body mass (BM, g). SCL and SCW were then used to calculate the size index (SCL × SCW, mm^2^). The size index was positively correlated with initial egg mass (r^2^ = 0.80, p = 0.02). An analysis of covariance (ANCOVA) with initial egg mass as the covariate and IT and clutch as the factors detected no significant differences in hatchling size index between IT treatments or among clutches (IT: p = 0.54; clutch: p = 0.70). An analysis of variance (ANOVA) with IT and clutch as the factors indicated that hatchlings incubated under cool IT conditions had significantly longer incubation periods (60.7 ± 0.6 day, mean ± SD) than hatchlings incubated under warm IT conditions (46.3 ± 2.1 day, mean ± SD) (IT: p = 0.004; clutch: p = 0.25).

### Swimming performance

Hatchlings mainly produce thrust while swimming using ‘powerstroke’ in which the forelimbs move simultaneously^28^. We calculated four measures for hatchling swimming performances: (1) mean thrust (millinewton: mN), (2) maximum thrust (mN), (3) time spent powerstroking (%), and (4) number of powerstrokes (strokes/min). Swimming performance was measured by the method previously described^18^. In addition, one IT group was divided into a warm WT group (30 °C) and a cool WT group (27 °C) to assess the effects of WT on the swimming performance. WTs consisted of the minimum and maximum WT during hatching season in waters adjacent to our field study area.

**Figure 1:**
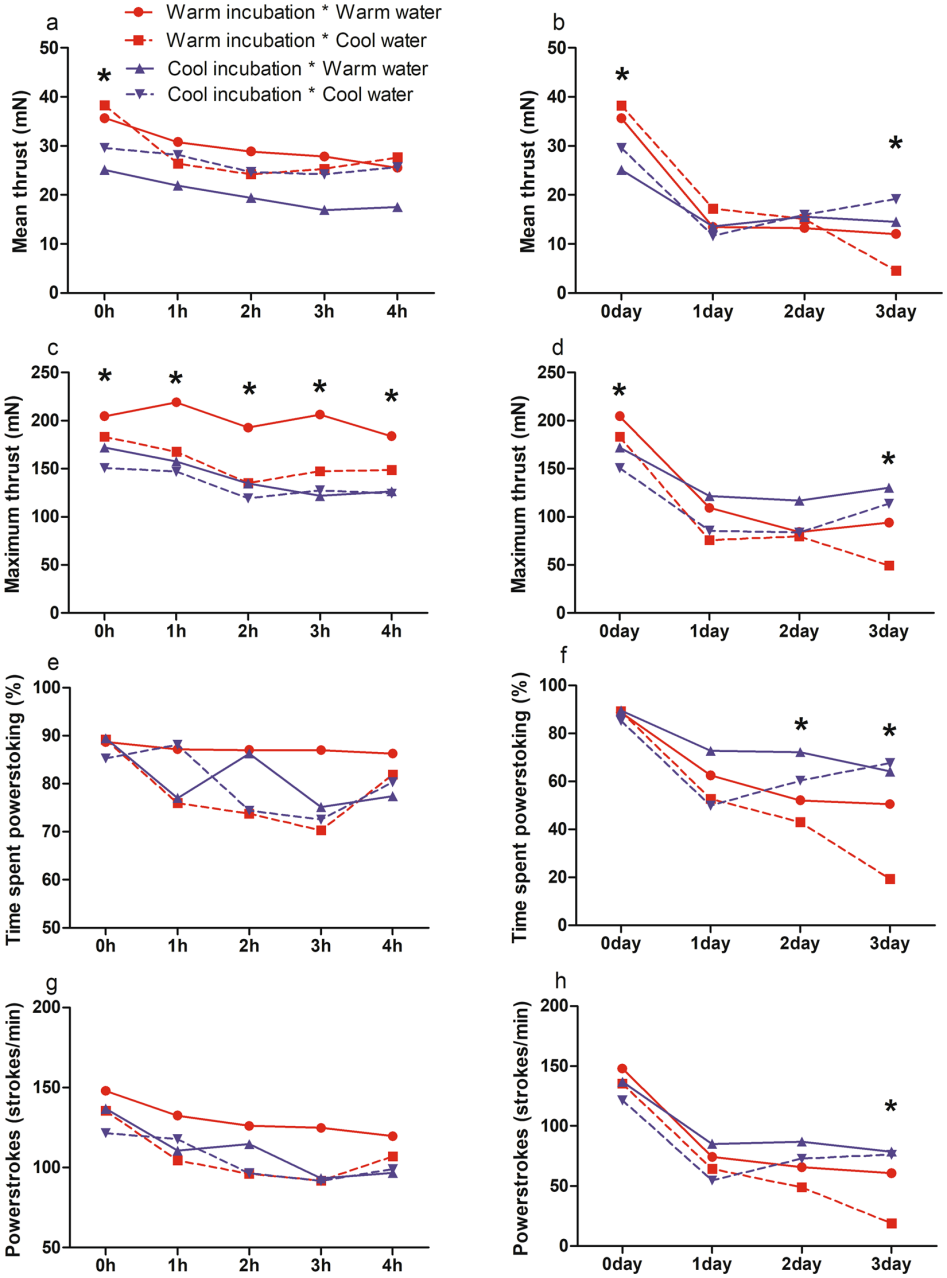
The effect of incubation temperature and water temperature on the swimming performance of loggerhead sea turtles (n = 12 for each group). (**a**) Mean thrust from 0 h to 4 h and (**b**) 0 d to 3 d (72 h). (**c**) Maximum thrust from 0 h to 4 h and (**d**) 0 d to 3 d (72 h). (**e**) Time spent powerstroking from 0 h to 4 h and (**f**) 0 d to 3 d (72 h). (**g**) Number of powerstrokes from 0 h to 4 h and (**h**) 0 d to 3 d (72 h). Results are expressed as means; asterisk indicates that the effect of incubation temperature on the swimming performance was statistically significant.

All four measures declined over the experimental period (Fig. 1). Repeated measures ANOVA with IT, WT, and clutch as factors indicated that although there was no significant effect of IT and clutch on swimming performance, there were significant interactions between time × IT and time × clutch. At 0 h, mean thrust and maximum thrust in the warm IT group were significantly greater than in the cool IT group (mean thrust: p = 0.008, maximum thrust: p < 0.05) (Fig. 1a,c). This IT effect continued through the first to 4 h of maximum thrust (1 h: p = 0.01, 2 h: p = 0.03, 3 h, p = 0.002, and 4 h, p = 0.01) (Fig. 1c), but there was no effect on mean thrust (Fig. 1a). This IT effect was reversed after 48 h, however; time spent powerstroking was greater among hatchlings in the cool IT group than those in the warm IT group at 48 h (p = 0.04) (Fig. 1f), and all four measures were higher among hatchlings in the cool IT group than in the warm IT group at 72 h (mean thrust: p = 0.02, maximum thrust: p = 0.003, time spent powerstroking: p < 0.001, number of powerstrokes: p = 0.002) (Fig. 1b,d,f,h). In addition, several of the measures were significantly higher in hatchlings exposed to warm WT conditions over the course of the experimental period (mean thrust: p = 0.56, maximum thrust: p = 0.01, time spent powerstroking: p = 0.13, number of powerstrokes: p = 0.04) (Fig. 1).

### Growth rate

**Figure 2:**
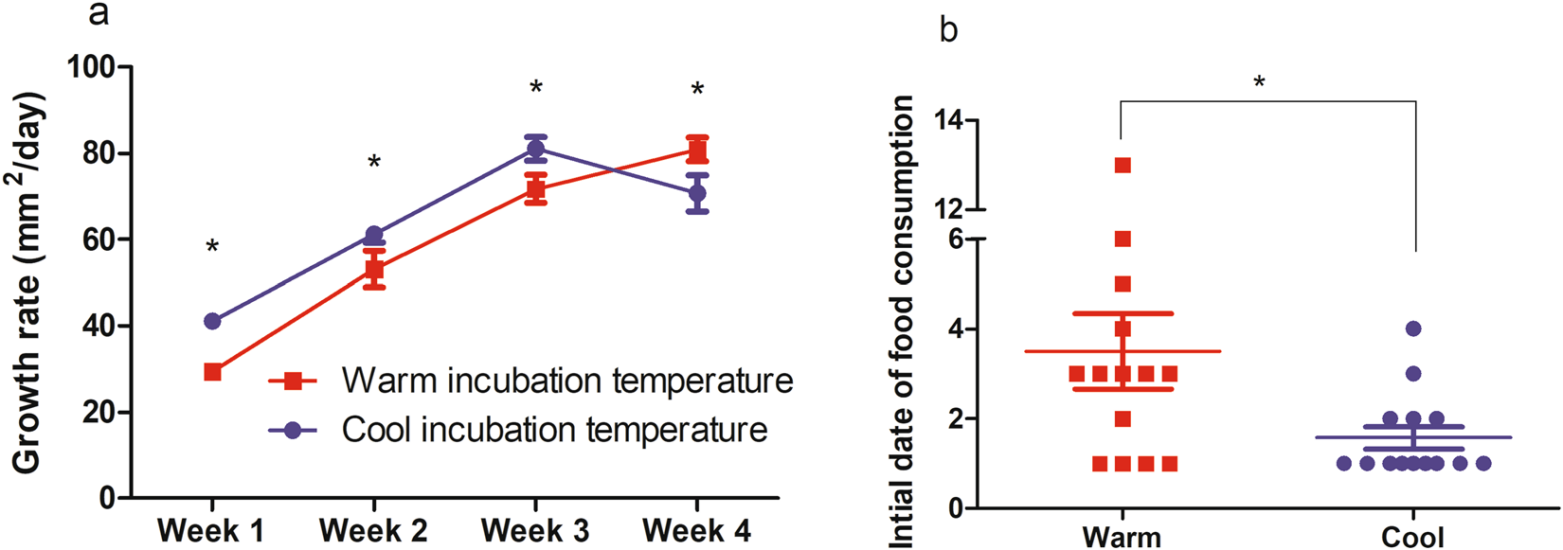
Growth rate and initial date of food consumption of loggerhead sea turtles (n = 14 for each group). Size index (SCL × SCW) was used for the size parameter. (**a**) Weekly change of daily growth rate between the incubation temperature groups. Results are expressed as mean ± SEM; asterisk indicates a significant difference between columns (p < 0.05). (**b**) The initial date of food consumption in the incubation temperature groups. Results are expressed as mean ± SEM; asterisk indicates a significant difference between columns (p < 0.05).

To assess the effect of IT on hatchling growth rate, the size indice of reared hatchlings were measured weekly over 4-week period. A rearing period of four weeks was followed because loggerhead sea turtle hatchlings depart from the neritic zone 5–30 days after hatching^12^. Repeated measures ANOVA with IT and clutch as factors indicated that the daily growth rate during each week (mm^2^/day) increased significantly at each measuring period among hatchlings in the warm IT group (p < 0.001), whereas daily growth rate decreased at week 4 among hatchlings in the cool IT group (p < 0.001) (Fig. 2a). Although the effect of IT and clutch on hatchling growth rate was not significant (IT: p = 0.12, clutch: p = 0.63), the interactions between time × IT (p < 0.001) and time × IT × clutch (p < 0.001) were found to be significant. Simple main effects analysis for the interaction between time × IT indicated that the IT effect was significant at all times of measurement (week 1: p = 0.005, week 2: p < 0.05, week 3: p = 0.02, week 4: p = 0.01). From week 1 to week 3, hatchlings in the cool IT group had higher growth rates than hatchlings in the warm IT group, but this was reversed at week 4 (Fig. 2a). Size index at the time of initial measurement was not correlated with growth rate during the measuring period (r^2^ = 0.01, p = 0.55).

Interestingly, the initial date of food consumption in the warm IT group varied (3.5 ± 3.1 days) (mean ± SD) and occurred significantly earlier in the cool IT group (1.6 ± 0.9 days) (mean ± SD) (p = 0.03) (Fig. 2b). Moreover, initial date of food consumption was significantly negatively correlated with growth rate at week 2 and 3 but not at week 1 and 4 (week 1: spearman r = −0.21, p = 0.27; week 2: spearman r = −0.48, p = 0.01; week 3: spearman r = −0.50, p = 0.007; week 4: spearman r = 0.13, p = 0.52).

### Blood glucose concentrations

**Figure 3:**
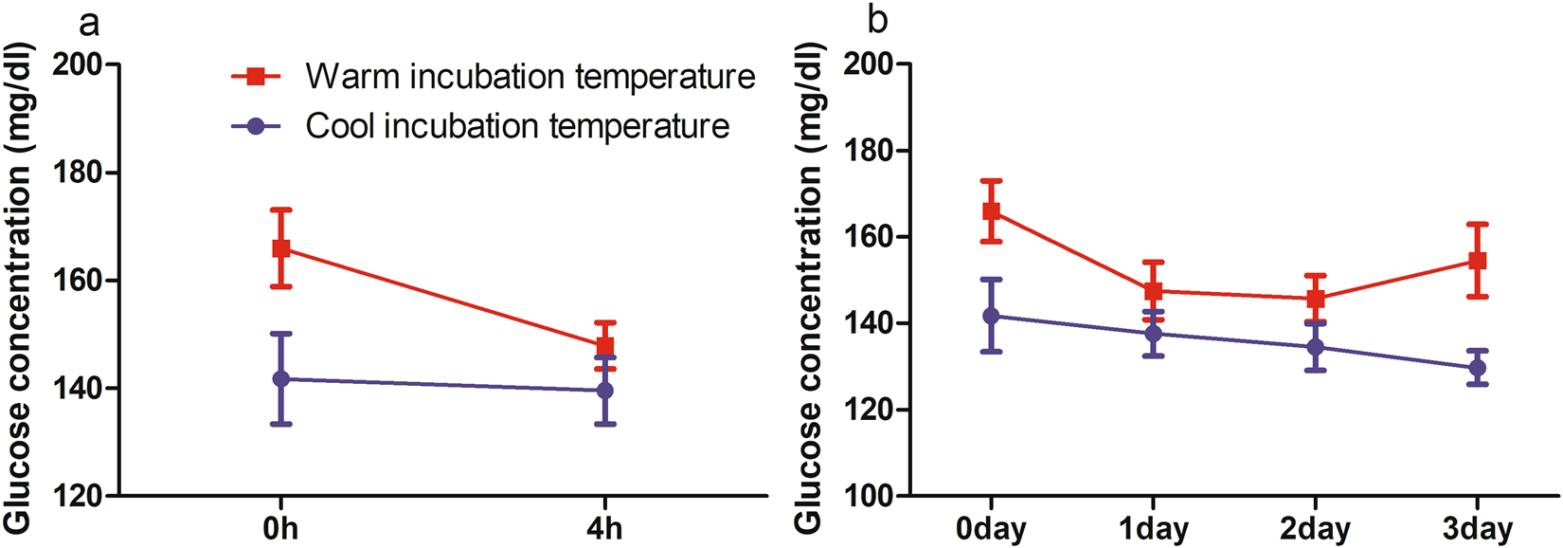
The effect of incubation temperature on blood glucose concentration of loggerhead sea turtles from (**a**) 0 h to 4 h and (**b**) 0 d to 3 d (72 h) (n = 7–8 at each sampling point for each IT group). Results are expressed as mean ± SEM.

To examine whether IT affects blood glucose concentration, we measured hatchling blood glucose concentration at 0, 4, 24, 48, and 72 h after measuring their size. An ANOVA with IT, clutch, and time as factors revealed that blood glucose concentrations were approximately 1.1 times higher in the warm IT group than hatchlings in the cool IT group (p < 0.001) (Fig. 3a,b). Blood glucose concentrations differed significantly between the clutches (p < 0.001), but not sampling period (p = 0.11).

## Discussion

We analysed the effect of IT and WT on several performance traits that potentially affect the survival of loggerhead sea turtle hatchlings during the post-hatchling dispersal period (frenzy and post-frenzy periods) such as body size, swimming performance, growth rate, and blood glucose concentrations. We did not find any IT effect on the size index. Previous studies have shown that hatchlings incubated under cooler IT tended to be larger^16,19,20,22,29–32^, because cooler IT prolongs the incubation period when the yolk is converted to tissue^19^. However, one study that examined green sea turtle clutches that were divided and incubated at different temperatures in the field suggested that the maternal factor (initial egg mass) is more important for determining the size of hatchlings than environmental factors (i.e., IT)^20^. As such, although IT may affect the size of hatchlings, it may not be as in determining hatchling size as other factors, such as initial egg mass.

From 0 h to 4 h, maximum thrust was higher in the warm IT group than in the cool IT group, suggesting that IT is involved in hatchling muscle strength during the frenzy period. However, although mean thrust was also significantly higher in the warm IT group at time of the initial measurement, significance disappeared after 1 h. This suggests that IT may not greatly influence swimming performance during the frenzy period in loggerhead sea turtles. In contrast, time spent powerstroking was higher in the cool IT group than in the warm IT group at 48 h, and all four measures of swimming performance were significantly higher in the cool IT group at 72 h. Therefore, IT plays a role not only in initial swimming performance but also in sustained swimming performance during their post-frenzy period. Although the mechanism explaining how IT influences hatchling swimming performance is unknown, the involvement of IT in the muscle fibre type (i.e., fast twitch fibre and slow twitch fibre) development has recently considered^33^. The different ITs have different effects on initial and sustained swimming performance may be key to elucidate the relationship between IT and muscle fibre development.

Previous studies have shown that hatchlings incubated under warm IT conditions had more remaining yolk and consumed less energy during embryonic growth than hatchlings incubated under cooler IT conditions due to shorter incubation period^19,25^, and thus, hatchlings incubated under warm IT possibly have superior energy reserves after hatching^19^. In the present study, we found that blood glucose concentrations, a candidate energy source for swimming performances^34,35^, was higher in hatchlings in the warm IT group than in the cool IT group, offering further support for this hypothesis. Although glucose is used as energy source for hatchling swimming performance^34,35^, blood glucose concentration did not decrease notably between 0 h and 4 h. Conceivably, blood glucose could be supplied to the bloodstream from the remaining yolk, and/or glycogen breakdown in the liver when blood glucose is used for swimming. During the experimental study period (0 h to 72 h), blood glucose concentrations did not change, suggesting that hatchling swimming performance during post-frenzy periods were fuelled by another energy source, such as fat. Exploring the relationship between swimming performance and the various potential energy sources is key to reveal the mechanism of swimming locomotor activity.

Swimming performance was also affected by WT, with hatchlings in the warm WT group exhibiting superior swimming performances traits than hatchlings in the cool WT group. Such WT effect has been reported previously in green sea turtle hatchlings^16^. Because reptiles are ectotherms, their heart rates are dependent on environmental temperature^36^, and therefore, warm WT could enhance hatchling metabolic rate and consequently, improve swimming performance during the frenzy and post-frenzy periods.

Hatchlings in the cool IT group exhibited higher growth rates than hatchlings in the warm IT group over the first three weeks, but this was reversed at week 4. This phenomenon would be ‘catch-up growth’, in which growth rates reach optimal level if growth is limited during development^37^, as documented in other species^37^. A previous study of growth rates in loggerhead sea turtle hatchlings in the North Atlantic revealed that hatchlings born in northern latitudes and during the early nesting season had higher growth rates over several weeks^27^. These hatchlings experienced cooler IT conditions^27^, suggesting that IT might play a role in promoting hatchling growth rate; however, genetic factors and/or maternal nutrition during the nesting season also likely contributed to hatchling growth rates. Our results also suggested that IT plays a role in the growth rate of loggerhead sea turtle hatchlings. Furthermore, we found that clutch had no significant effect on growth rate, indicating that genetic and/or maternal factors may not greatly influence growth rate.

In the present study, hatchlings in the cool IT group started consuming food earlier than in the warm IT group. Moreover, a significant negative correlation was detected between initial date of food consumption and growth rate at week 2 and 3. Studies have shown that both chickens (*Gallus gallus*) and turkeys (*Meleagris gallopavo*) exhibit high growth rates in response to earlier feeding^38,39^. This suggests that the initial date of food consumption could be important for hatchling growth rate. Why then, did hatchlings in the cool IT group begin consuming food earlier? Generally, hunger is stimulated when the brain detects low blood glucose concentrations^40^. Lower blood glucose concentrations of the hatchlings in the cool IT group up to 72 h may occur because the cool IT prolonged the incubation period, during which hatchlings consumed more energy compared to those in warm IT conditions. Thus, prolongation of the incubation period under cool IT conditions may stimulate hunger, earlier food consumption, and subsequently, higher growth rates observed during the initial three weeks.

Such IT effects are likely to be involved in the survival of sea turtle hatchlings in nature. Our results suggest that loggerhead sea turtle hatchlings incubated under cooler IT may be able to sustain their swimming locomotor activity, and can leave the neritic zone more rapidly, improving survival. Moreover, although this may only be the case for our study area, since the WT during the early hatching season (hatched from nests in the early nesting season) was warm and WT during the late hatching season (hatched from nests in the later nesting season) was cool, the swimming activity of hatchlings from nests in the early hatching season (warm WT) is potentially higher than that in the late nesting season (cool WT). Furthermore, if food availability is rich when hatchlings leave the neritic zone, then hatchlings incubated under cooler IT conditions grow more rapidly during the initial three weeks following the earlier initiation of food consumption, improving their ability to escape from gape-limited predators. In addition, terrestrial locomotor performance is reported to be higher in hatchlings incubated under cooler IT conditions in the field nest^18,20^. Because sea turtle eggs laid in the early and late nesting seasons are generally incubated under cool IT conditions (producing males) and warm IT conditions (producing females), respectively^41–53^, taken together, we suggest that hatchling survival rates during early life stages will differ by seasons as well as sexes. Such differences might account for why the sex ratio of juveniles is less female-biased than that of hatchlings in some populations^54–56^. Notably, sea turtles have survived many changes in climatic conditions over the past hundred million years^57^, suggesting that such sex differences of survival during early life stages might be one of the adaptive mechanisms to adjust the sex ratio at later life stages in response to changes in environmental condition. In the present study, eggs were incubated at a constant temperature and hatchlings were well fed, dissimilar from typical field conditions. Further experiments that include evaluations of swimming performance of hatchlings in the field and the growth rates of non-fed hatchlings are needed to assess differences in survival between male and female hatchlings under natural conditions.

As increasing rates of feminisation associated with climate change have been anticipated^2–6^, cooling IT methods such as shading and watering are considered to be viable conservation strategies^5,7–9^. However, our results indicated that altering IT indirectly influences the survival of loggerhead hatchlings by affecting swimming performance traits and growth rates, which in turn may influence predator avoidance. Moreover, cooling IT methods to FPT nests would extend the incubation period by approximately two weeks. Using our study area as an example, WT changes approximately 2 °C within 2 weeks, potentially affecting swimming performance. Artificial manipulation of IT should then be carefully considered. Whether and how sea turtles adapt to a warming world is still largely unknown, and moreover, the extent and direction of climate change will differ among regions. Examining potential conservation strategies under future climate change is of critical importance and, where possible, must be conducted at global scales.

Although we demonstrated that incubation and/or water temperatures had significant effects on several factors that influence hatchling survival during the dispersal phase, our experiments involved small sample sizes. While our results must be taken with some caution, and further research is needed, they provided a strong indication that artificial manipulation of incubation temperature could affect hatchling performance during the dispersal phase, and consequently, overall survival. Our results shed further light on the complexities of conservation strategies for sea turtles faced with changing environmental conditions due to global climate change.

## Materials and Methods

Field surveys, animal care, animal use, and treatment were performed in accordance with the guidelines of the Animal Ethic Committee of Kochi University, and the protocol of the study was approved by this committee.

Wild turtle eggs were collected with the permission of the Kochi Prefecture Government, Department of Forestry and the Environment. Wild loggerhead turtles that laid eggs on an artificial beach at the Port of Nagoya Public Aquarium (PNPA) were originally captured with permission from the Shizuoka, Mie, and Kagoshima Prefecture Governments.

### Sampling procedure

To collect loggerhead eggs, we patrolled Kochi Beach (Kochi Prefecture), a known rookery for this species in the North Pacific^18^, daily during the nesting season. From discovered nests we collected about 80 eggs, which were stored in a bucket and transported to the laboratory by car (approximately 25 min from sampling to arrival at the laboratory). Two clutches were collected from Kochi Beach, and one clutch was collected from the artificial beach in the PNPA. Four days after nesting at PNPA, the clutch was sampled, stored in a styrofoam box, and transported to the laboratory via train and auto. Conditions in the styrofoam box were frequently monitored, and constant temperature and humidity were maintained. Transporting the samples to the laboratory required approximately 6 h. Upon arrival at the laboratory, initial egg masses (n = 10 eggs per clutch) were weighed, and the clutch was stored in an incubator (Rcom Reptile 90 Pro, Rcom, Korea).

### Egg incubation

The clutches were equally divided into two IT groups and stored in the incubators, one of which was set to 31 °C (warm IT) and the other to 27.5 °C (cool IT). Incubators were placed in a dark room; lids were covered with black cloth and the bottom of the incubator was covered with sand collected at Kochi Beach. Eggs were covered with wet bog moss to maintain humidity. We monitored the temperature in the incubator daily with an infrared radiation thermometer (FLIR TG165, FLIR system, USA).

Hatching was defined as a head and a flipper emerging from the shell^58^ and was determined with a night vision scope (NVMT Spartan 2 × 24, Yukon Advanced Optics Worldwide, Lithuania). Hatchling SCL, SCW, and BM were initially measured four days after hatching. Swimming performances, growth rates, and blood glucose concentrations were subsequently assessed in these hatchlings.

### Swimming performance

We randomly selected six hatchlings from the warm and the cool IT groups and measured their swimming performances. Hatchlings swam individually in a plastic tank and were fitted with a harness, which was attached via monofilament polyethylene string to a force transducer (MLTFO 50/ST, ADInstruments, Australia) connected to a data acquisition system (Power Lab 8/35 ADInstruments, Australia) programmed to record the force 40 times per second^18^. To assess whether WT affected swimming performance, we divided the IT groups into two groups, in which three hatchlings swam in 30 °C (warm WT) or three hatchlings swam in 27 °C (cool WT). Measurements were taken continuously from 0 h to 4 h, then at 24, 48, and 72 h after measurement of body size. Mean thrust was calculated by averaging all sampling points during 10 min of the recording period. Maximum thrust was averaged from the maximum values during 1 min intervals at 0–1, 5–6, and 9–10 min over a 10 min recording period. The time spent powerstroking and number of powerstrokes were based on the particular wave profile recorded on the analysis monitor when powerstroking was observed and averaged during 1 min intervals at 0–1, 5–6, and 9–10 min over a 10 min recording period. When untested, hatchlings were unfed and maintained in another tanks set at the same WT at which they swam.

### Growth rate

To measure growth rates, we reared seven hatchlings from the warm IT and cool IT groups from clutches laid at Kochi Beach for a period of 4 weeks. Hatchlings were individually reared in a glass tank and compartmentalized by plastic netting (25 cm × 25 cm × 25 cm) to prevent interactions. Rearing temperature was maintained at 30 °C using a heater with a thermostat, matched to sea temperature in our study area during the hatching period. When feeding, we transferred hatchlings to individual bowls to prevent the formation of feeding hierarchies and the deterioration of rearing water quality. Bait food consisted of a paste mixture composed of shell, shrimp, and squid. Hatchlings were fed for 1 h. After 30 min of the commencement of feeding, we checked remaining bait and added a sufficient amount when food levels were low. We measured SCL and SCW weekly and calculated the growth rate of size index using the equations:

$${\rm{D}}{\rm{a}}{\rm{i}}{\rm{l}}{\rm{y}}\,{\rm{g}}{\rm{r}}{\rm{o}}{\rm{w}}{\rm{t}}{\rm{h}}\,{\rm{r}}{\rm{a}}{\rm{t}}{\rm{e}}\,{\rm{a}}{\rm{t}}\,{\rm{w}}{\rm{e}}{\rm{e}}{\rm{k}}\,{\rm{n}}\,({{\rm{m}}{\rm{m}}}^{2}/{\rm{d}}{\rm{a}}{\rm{y}})=({{\rm{S}}}_{n}-{{\rm{S}}}_{n-1})/7$$

where *n* is the week number of rearing hatchlings and *S* is the size index (S_0_: initial measurement). Food consumption was defined to be when a hatchling picked at the bait at least twice in 10 s, and the initial date of food consumption was recorded.

### Blood glucose concentrations

We collected the blood of hatchlings from clutches laid at Kochi Beach, excluding that of hatchlings used in the swimming performance and rearing analyses. Hatchlings included in blood collection were maintained in a tank at 28 °C without feeding until blood collection was completed (72 h). We did not use hatchlings whose blood had already been collected. Approximately 500 μl of blood was collected from the dorsal cervical sinus using a 26 G needle (Termo, Japan) and a 1 mL syringe (Termo, Japan) at 0, 4, 24, 48, and 72 h after size measuring (n = 3–4 at each sampling time). Blood was poured into a heparinised tube (Fujifilm, Japan) and centrifuged at 3600 g for 10 min. Plasma was moved into another tube and stored at −80 °C until glucose measurements. Blood glucose concentrations were measured enzymatically using the glucose-oxidase method (Glucose CII-test, Wako Pure Chemical Industries, Japan) according to the manufacturer’s protocol.

### Statistics

The relationships between initial egg mass, size index and growth rates were analysed using linear regression. Spearman’s rank correlation test was used to test for correlations between growth rate and initial date of food consumption. The effects of IT on size was tested using ANCOVA, with initial egg mass as the covariate. The effects of IT on the incubation period, initial date of food consumption and blood glucose concentrations were statistically tested with ANOVA. Repeated measure ANOVA was used to test the effects of IT and WT on swimming performance and growth rates. Factors of ANCOVA and ANOVA have been described in the Results. In cases where a significant interaction was detected by ANOVA, we used a simple main effect analysis to confirm when the IT effect was significant. All statistical analyses were performed with the Graph Pad Prism5 (San Diego, CA, USA) and Excel Statistics 2012 (Tokyo, Japan), and p values < 0.05% were considered to be significant.

### Data availability

The data that support the findings of the present study are available from the corresponding author upon reasonable request.
